# The safe use of dating applications among men who have sex with men: a study protocol for a randomised controlled trial to evaluate an interactive web-based intervention to reduce risky sexual behaviours

**DOI:** 10.1186/s12889-020-08914-z

**Published:** 2020-05-27

**Authors:** Edmond P. H. Choi, Eric P. F. Chow, Eric Y. F. Wan, William C. W. Wong, Janet Y. H. Wong, Daniel Y. T. Fong

**Affiliations:** 1 School of Nursing, University of Hong Kong, 4/F, William M.W. Mong Block 21 Sassoon Road, Pokfulam, Hong Kong; 2 Melbourne Sexual Health Centre, Alfred Health, Melbourne, VIC Australia; 3 Central Clinical School, Monash University, Melbourne, VIC Australia; 4 Centre for Epidemiology and Biostatistics, Melbourne School of Population and Global Health, The University of Melbourne, Parkville, VIC Australia; 5 Department of Family Medicine and Primary Care, University of Hong Kong, Pokfulam, Hong Kong; 6 Department of Pharmacology and Pharmacy, University of Hong Kong, Pokfulam, Hong Kong

**Keywords:** Dating apps, Men who have sex with men, Risky sexual behaviours, Sexual health

## Abstract

**Background:**

Notably, both international and local studies have found a high prevalence of sexually transmitted infections (STIs) and risky sexual behaviours, such as condomless anal sex, substance misuse in conjunction with sex (‘chemsex’) and group sex, among men who have sex with men (MSM) dating application (app) users. Although the use of dating apps is an emerging sexual risk factor, little effort has been expended on the promotion of safe sex and good sexual health among the users of those apps. Therefore, the aim of the proposed study is to develop and evaluate the effectiveness of an interactive web-based intervention in improving the sexual health of MSM dating app users in Hong Kong.

**Methods:**

A two-armed randomised controlled trial will be conducted. Chinese MSM dating app users will be recruited and randomly allocated into either the intervention (*n* = 200) or control group (*n* = 200). Subjects in the intervention group will receive the web-based intervention containing interactive content that (1) encourages a positive attitude towards consistent condom use and HIV/STI testing and negative attitude towards chemsex and group sex; (2) positions condom use and regular HIV/STI testing as normative; and (3) targets improved perceived self-efficacy concerning condom use and negotiation and HIV/STI testing. The control group will receive only web-based information without sexual health components. Subjects in both groups will be evaluated at baseline and three and 6 months after baseline. The primary outcome will be the frequency of condomless anal sex in the past 3 months.

**Discussion:**

The proposed study will aid development of culturally relevant health promotion programmes aimed at minimising the potential harm of dating app use and promoting the sexual health of MSM dating app users. The web-based intervention, if found successful, will have important clinical and policy implications, as it can be adopted by the government and non-governmental organisations targeting MSM. Moreover, the proposed intervention can reach many MSM at relatively low cost, and thus has the potential to check the burgeoning HIV/STI epidemic among MSM in Hong Kong in a cost-effective manner.

**Trial registration:**

International standard randomized controlled trial number (ISRCTN) registry: ISRCTN16681863 registered on 28 April 2020.

## Background

### Use of smartphone dating applications (apps): a contemporary phenomenon

Advances in mobile technology now allow Internet access via smartphones. The growing popularity of smartphones and Internet access worldwide have led to the launch of a wide variety of location-based smartphone dating applications (apps), also known as geosocial networking apps that make use of the global positioning system (GPS) [1]. GPS technology allows these apps to connect users with fellow users in the immediate vicinity. Furthermore, dating apps allow users to create profiles comprising a self-introduction, photos and basic demographic information, as well as what they are seeking in a partner. Whilst some apps, such as Tinder and Skout, have been developed for both the homosexual and heterosexual populations; others apps such as Grindr and Jack’d specifically target homosexual and bisexual men [2]. Dating apps have attracted considerable public attention in recent years. According to the Pew Research Center, in 2015, 9% of adults in the United States reported having used a smartphone dating app at some point, which represented a threefold increase over 2013, when just 3% of the country’s adults reported such use [3].

Uses and gratification theory [4, 5] and affordance theory [6] have been employed to explain the motives for using dating apps [7–10]. The former posits that physical, social and psychosocial gratification needs induce people to use such apps [7, 11, 12]. The physical gratification sought through dating apps includes sexual pleasure and desire [12], whilst the social gratification includes love and romance [1]. Psychosocial gratification comprises a need for stimulation and validation of one’s sexual attractiveness [1]. In contrast, affordance theory suggests that mobility, proximity and immediacy are the motives for using dating apps [10]. In terms of mobility, users can access the apps anywhere at any time, as most people tend to carry their smartphones wherever they go. In terms of proximity, dating apps connect users with others in their immediate vicinity, and, in terms of immediacy, some dating app users have suggested that the apps facilitate fast and even immediate sexual encounters [10].

### Dating app use among men who have sex with men

There has been a shift in the way that men who have sex with men (MSM) seeking partners over the decades, moving from the traditional way (in person at gay bars or other venues) to the Internet and now to smartphone dating apps [13]. Most MSM today rely on dating apps to find sexual partners. A study conducted in Melbourne found that 71% of MSM meet sexual partners via dating apps [13]. There are several explanations for the popularity of dating app use for seeking sex among MSM. First, sexual minorities constitute a small group relative to the heterosexual population, and it is thus more difficult for them to encounter one another in daily social activities [14]. Online environments, in contrast, can serve as a hub, making it easier for sexual minorities to gather and meet. Second, it can be embarrassing for sexual minorities to initiate relationships in offline contexts because of stigmatisation and discrimination [14]. Online environments provide a protected platform to meet potential partners without exposing too much about one’s identity and sexual orientation [15]. The pervasive use of online media to find sexual partners among MSM indicates that it may be useful to include such media in human immunodeficiency virus (HIV) and sexually transmitted infection (STI) prevention activities [16–19]**.**

### The use of dating apps: an emerging risk factor in sexual health

The use of dating apps is considered a factor leading to the rapid spread of STIs, including HIV [20, 21]. In Hong Kong, it has been reported that it is not uncommon for dating apps to be employed in search of sexual activities [22]. A report also claimed that the abundance of dating apps has exacerbated the problem of recreational drug use during sexual contexts (known as ‘chemsex’) [23]. Furthermore, there has been a significant increase in police reports of sexual assaults and violence related to dating app use in the United Kingdom [24]. Several empirical studies have explored the association between dating app use and risky sexual behaviours [25, 26].

A systematic review summarising 13 articles on the use of dating apps and associated risky sexual behaviours among the lesbian, gay, bisexual and transgender populations, which found that dating app users tended to engage in more high-risk sexual behaviours than nonusers, including having sex with multiple partners and engaging in condomless anal sex [26]. This review affirms the need to promote safe sex and positive sexual health messages to dating app users*.* A study was conducted among university students in Hong Kong found that dating apps users were more likely than nonusers to have multiple sexual partners and condomless sex with more sexual partners, use condoms inconsistently, engage in casual sex, and sexual abuse [27–29]. Another study on MSM dating app users in Hong Kong found that about 30% of participants had had more than three sexual partners, 25% had engaged in condomless anal sex with casual partners, 20% had had condomless anal sex with internal ejaculation, 15% had used alcohol in conjunction with sex in the past 6 months, 13% had had group sex, 8% had had chemsex [30]. These alarming findings indicate the urgent need for interventions to encourage better and safe sexual health in Hong Kong dating app users.

### Efficacy, feasibility and acceptability of interactive web-based interventions

A Cochrane Review of interactive computer-based interventions (ICBIs) aimed at sexual health promotion concluded that ICBIs are effective for learning about sexual health [31]. ‘Interactive intervention’ is defined as intervention comprising components that require user contributions (e.g. completing knowledge tests, entering personal data and making choices) to produce tailored material and feedback that is personally relevant to users [32]. Furthermore, ICBIs have also been reported to exert positive effects on self-efficacy, safe sex intention and sexual behaviours [31]. A systematic review indicated that computer-based interventions are effective in increasing condom use and reducing sexual activity and the number of sexual partners [33]. Another systematic review on HIV prevention in the MSM population recommended eHealth interventions (including web-based interventions) to reduce HIV risk behaviours [34].

Web-based interventions offer several advantages. First, sexual health is considered an embarrassing and taboo topic in Chinese culture, and web-based interventions afford greater anonymity and privacy than other interventions. Second, participants can access such interventions at their convenience. Third, it is easy for web-based interventions to record the frequency and duration of intervention access. Fourth, web-based platforms facilitate data collection. Fifth, educational content on the web can be easily updated. Finally, the dissemination of web-based interventions is fast and relatively cheap. In terms of acceptability, one study found that 70% of MSM dating app users were willing to participate in a computer-based sexual health intervention [35]. Therefore, a web-based intervention is recommended for the proposed study.

### Proposed theoretical framework: theory of planned behaviour

According to the theory of planned behaviour (TPB), behaviour is primarily determined by intention and perceived control [36], and attitudes, perceived norms and behavioural control are theorised to predict intention. A meta-analysis reported that theory-based interventions designed to reduce risky sexual behaviours are more effective than those without a theoretical model [37]. According to various studies and meta-analyses, the TPB is an effective and appropriate model for predicting sexual behaviours [38, 39]. For example, a meta-analysis of 96 datasets supported the use of the TPB to predict future condom use [39]. Moreover, a more recent meta-analysis of 8 studies (2 cross-sectional studies and 6 prospective studies) published in 2016 suggested that the TPB is a useful model for explaining condom use behaviour among MSM [38]. The findings of this meta-analysis suggest that the TPB construct relationships are strong when applied to condom use among MSM [38]. Another meta-analysis of 35 intervention studies also supported the TPB’s use as a theoretical framework for designing interventions to change sexual risk behaviours [40]. Therefore, the TPB is recommended for this study.

### Aims and objectives

The aim of this study is to evaluate the effectiveness of the interactive web-based intervention in improving sexual health of dating apps users. Its three specific objectives are: (i) to reduce risky sexual behaviours (frequency of condomless anal sex, group sex and chemsex); (ii) to enhance users’ self-efficacy in and attitudes towards condom use; and (iii) to increase the frequency of HIV and STI testing.

### Hypotheses

It is hypothesised that, men receiving the web-based intervention are more likely to exhibit (i) fewer risky sexual behaviours, (ii) better efficacy in and positive attitudes towards condom use, and (iii) more HIV and STI testing, compared to men not receiving the web-based intervention.

## Methods

### Study design

It is a two-armed non-blinded randomised control trial (RCT) design (with a 6-month follow-up period) will be adopted for this study.

### Participants and sample size justification

A total of 400 subjects will be recruited for the RCT. Based on an effect size of 0.3 (as reported in meta-analysis [33]), it has been determined that the sample size for each group should be 175 (i.e. 350 in total) to achieve 80% power in detecting a between-group difference via an independent t-test at a 0.05 significance level. Assuming an estimated 12.4% attrition rate (as reported in an RCT in a similar setting [41]), a total of 400 subjects (i.e. 200 subjects in each group) is necessary.

To be eligible for inclusion in the RCT, individuals must be (i) MSM, (ii) cis male, (iii) aged 18 or above, (iv) current dating app users, (v) HIV-negative, (vi) sexually active, and (vii) able to read and understand Chinese.

### Sampling frame

Multiple sources of recruitment will be employed. First, local non-governmental organisations that target the MSM population will help to recruit participants. Second, given the high prevalence of dating app use among university students found in the previous study [28], mass university emails and on-campus posters will be used to recruit potential participants. Third, promotional materials will be posted on social media and online forums targeting MSM. Fourth, direct recruitment via dating apps will take place. Finally, snowballing will be used, with enrolled participants asked to invite potentially interested friends to join the study.

### Online enrolment and consent

Participants will enrol in the trial through the intervention website. A screening questionnaire will be administered to ensure participant eligibility. Eligible participants will then be asked to sign an electronic consent form and provide contact information. After these preliminary steps, participants will be requested to complete a web-based baseline questionnaire.

### Randomisation and allocation concealment

After completion of the baseline questionnaire, participants will be randomly assigned to either the intervention group or control group via computer-generated block randomisation (with blocks of size 4) on a 1:1 randomisation ratio; no stratification will be applied. Participants will be automatically guided to the web content associated with their allocation.

### Intervention group

A participatory design approach is used to develop the intervention. A qualitative study has been conducted to understand dating app users’ experience of using dating apps and engaging in risky sexual behaviours and exploring what elements of sexual health education are likely to be attractive and engaging to this population. The findings of the qualitative study will inform the intervention development.

The theoretical framework of the intervention will be based on the TPB. The aims of the intervention are to: (i) encourage a positive attitude towards consistent condom use and HIV/STI testing, and negative attitude towards chemsex and group sex; (ii) position condom use and regular HIV/STI testing as normative; and (iii) improve perceived self-efficacy concerning condom use and negotiation and HIV/STI testing. The intervention is expected to feature: (i) interactive components, and (ii) other educational materials.

### Control group

The control group will participate in a web-based intervention with no sexual health information.

Participants in both groups will be afforded unlimited access to their allocated content over the 6-month study period, but that content will be available only to participants who log in with a registered email address and password. After the study period, the content accessed by the intervention group will be made available to the control group subjects as well.

### Study outcomes

The study outcomes will be measured in both groups at baseline (T0) and at 3 months (T1) and 6 months (T2) after baseline. The primary outcome will be the frequency of condomless anal sex in the past 3 months, based on the recommendation of a systematic review of 56 studies [42]. The secondary outcomes will be the frequency of group sex and chemsex, self-efficacy and attitudes toward condom use, and frequency of HIV and other STI testing.

Data on these outcomes will be collected and evaluated as follows.

- Frequency of condomless anal sex in the past 3 months is measured by a self-reported item at baseline, 3-month follow-up and 6-month follow-up.
- Frequency of group sex in the past 3 months is measured by a self-reported item at baseline, 3-month follow-up and 6-month follow-up.
- Frequency of chemsex in the past 3 months is measured by a self-reported item at baseline, 3-month follow-up and 6-month follow-up.
- Self-efficacy in condom use is measured by the validated traditional Chinese version of the Condom Self-Efficacy Scale at baseline, 3-month follow-up and 6-month follow-up [43].
- Attitudes towards condom use is measured by the UCLA Multidimensional Condom Attitudes Scale at baseline, 3-month follow-up and 6-month follow-up [44].
- Frequency of HIV testing in the past 3 months is measured by a self-reported item at baseline, 3-month follow-up and 6-month follow-up.
- Frequency of STI testing in the past 3 months is measured by a self-reported item at baseline, 3-month follow-up and 6-month follow-up.

Finally, participants’ sociodemographic characteristics (e.g. age and education level), sexual histories (e.g. age of sexual initiation), reported use of pre-exposure prophylaxis and their patterns of dating app usage will be collected.

At 6-month follow-up (T2), participants will be asked whether they find the web-based information useful and are satisfied with the web-based information.

Table 1 shows the SPIRIT diagram for the schedule of enrolment, interventions, and assessments.

**Table 1:** SPIRIT diagram for the schedule of enrolment, interventions, and assessments

	Study period				
	Enrolment	Allocation	Post-allocation		Close-out
Timepoint	***-t***_***1***_	0	***T0***	***T1***	***T2***
**Enrolment:**					
**Eligibility screen**	X				
**Informed consent**	X				
**Allocation**		X			
**Interventions:**					
**Intervention group**			X	X	
**Control group**			X	X	
**Assessments:**					
**Primary outcome and secondary outcomes**^a^	X				
**Primary outcome and secondary outcomes**^a^				X	X

Note: The study outcomes will be measured in both groups at baseline and at 3 months (T1) and 6 months (T2) after baseline

^a^ The primary outcome is the frequency of condomless anal sex in the past 3 months. The secondary outcomes are the frequency of group sex and chemsex, self-efficacy and attitudes toward condom use, and frequency of HIV and other STI testing

### Data analysis

The continuous outcomes in the intervention and control groups at the 3- and 6-month follow-ups will be evaluated using an independent t-test, and then compared using a linear mixed-effects model with the intervention group as the covariate. Subject as random effect will be included in the model if significant. In case of a baseline imbalance, additional analysis will be performed, with adjustment of the variables found to be imbalanced. The intention-to-treat principle will be adopted, and all study subjects will be included in the analysis as randomised. The baseline-observation-carried-forward approach will be used for missing values at the 3- and 6-month follow-ups. Model adequacy will be verified by examining the standardised residuals for normality and constant variance.

## Discussion

The study is significant. Given the popularity of dating app use, and its associated sexual risks, interventions promoting safe sex practices among dating app users are urgently needed. Besides, HIV infection is also alarming in Hong Kong. HIV cases in Hong Kong hit the record high recently, with more young men infected. The number of new HIV cases increased from 513 in 2012 to 725 in 2015 and 681 in 2017. Homosexual contact is the major route of transmission. In 2017, more than 60% caught the HIV through sex between men, compared with an average of 40% from 1984 to 2015. The above local data delineate that HIV infection resulting from sex between men accounts for the majority of acquired HIV diagnoses in Hong Kong.

A literature review and meta-analysis suggest that peer involvement in web-based sexual health interventions improves knowledge, attitudes and, to some degree, safe sex behaviours [45]. To the best of our knowledge, this will be the first interactive web-based intervention to specifically target MSM dating app users. Unlike most health interventions, which are developed exclusively by healthcare providers, the proposed intervention will be developed by both healthcare providers and study participants (dating app users) using a participatory design approach. The acceptability and feasibility of this client-oriented intervention are thus assured. Ultimately, the intervention is expected to improve the sexual health of MSM. Finally, although tremendous efforts have been expended globally to prevent HIV and STIs among MSM, this population is still disproportionately affected by both. The proposed intervention will contribute to global HIV and STI prevention strategies.

## Abbreviations

Apps: applications
STI: Sexually transmitted infections
HIV: Human immunodeficiency virus
MSM: Men who have sex with men
ISRCTN: International standard randomized controlled trial number
GPS: Global positioning system
ICBIs: Interactive computer-based interventions
TPB: Theory of planned behaviour
RCT: Randomised control trial
